# Distinct miRNA Gene Expression Profiles Among the Nodule Tissues of Lung Sarcoidosis, Tuberculous Lymphadenitis and Normal Healthy Control Individuals

**DOI:** 10.3389/fmed.2020.527433

**Published:** 2020-10-16

**Authors:** Ya-bin Zhao, Wei Li, Qin Zhang, Yan Yin, Chuan-jia Yang, Wen-xiang Xu, Jian Kang, Rui-qun Qi, Gang Hou

**Affiliations:** ^1^Department of Respiratory Medicine, First Hospital of China Medical University, Shenyang, China; ^2^Department of Respiratory Medicine, The Third People's Hospital of Hubei Province, Wuhan, China; ^3^Department of Thyroid Surgery, Shengjing Hospital of China Medical University, Shenyang, China; ^4^Department of Pathology, Shenyang Thoracic Hospital, Shenyang, China; ^5^Department of Dermatology, First Hospital of China Medical University, Shenyang, China; ^6^Department of Pulmonary and Critical Care Medicine, Center of Respiratory Medicine, National Clinical Research Center for Respiratory Diseases, China-Japan Friendship Hospital, Beijing, China

**Keywords:** sarcoidosis, tuberculous lymphadenitis, MicroRNAs, diagnosis, lymph nodes

## Abstract

**Background:** Sarcoidosis and tuberculosis share similarities in clinical manifestations and histopathological features. We aimed to identify the microRNA (miRNA) profiles of the lymph nodes of individuals with sarcoidosis and of those with tuberculous lymphadenitis to investigate the value of miRNAs in the differential diagnosis of sarcoidosis and tuberculous lymphadenitis.

**Methods:** The miRNA profiles of the lymph nodes of individuals with sarcoidosis, those with tuberculous lymphadenitis (TBLN) and controls were detected by miRNA microarray analysis in the age- and sex-matched development group of the controls (*n* = 3), patients with TBLN (*n* = 3) and patients with sarcoidosis (*n* = 3), and the results were validated by quantitative real-time polymerase chain reaction in the validation group of the controls (*n* = 30), TBLN (*n* = 30) and patients with sarcoidosis (*n* = 31). The relationship between miRNA expression and the clinical parameters of sarcoidosis was analyzed.

**Results:** miR-145, miR-185-5p, miR-301, miR-425-5P, miR-449b and miR-885-5P were differentially expressed between individuals with sarcoidosis and controls (*P* < 0.0001, *P* < 0.0001, *P* = 0.0008, *P* = 0.0002, *P* = 0.0018, and *P* < 0.0001, respectively), and the same six miRNAs were differentially expressed between individuals with tuberculous lymphadenitis and controls (*P* = 0.0002, *P* = 0.0004, *P* = 0.0238, *P* = 0.0006, *P* = 0.0149, and *P* = 0.0045, respectively). miR-185-5p was differentially expressed between individuals with tuberculous lymphadenitis and those with sarcoidosis (*P* = 0.0101). The area under the receiver operating characteristic curve calculated for miR-185-5p was 0.6860, and the sensitivity and specificity of miR-185-5p for the differential diagnosis of sarcoidosis from TBLN were 61 and 80%, respectively. The levels of miR-145, miR-301, miR-425-5P, and miR-885-5P were positively correlated with CD4+/CD8+ T lymphocytes in bronchoalveolar lavage fluid.

**Conclusions:** miRNAs in lymph nodes show similar expression patterns between individuals with sarcoidosis and those with tuberculous lymphadenitis, which were experimentally selected. miR-185-5p in the lymph nodes can be used as an auxiliary marker for the differential diagnosis of sarcoidosis and tuberculous lymphadenitis.

## Introduction

Sarcoidosis and tuberculosis (TB) are chronic inflammatory diseases that share similarities in clinical manifestations and histopathological features (1, 2). The differential diagnosis of sarcoidosis and TB is challenging (3), especially in countries with a high TB burden (4, 5). The diagnosis of these two diseases usually depends on pathology and Mycobacterium tuberculosis (MTB) culture. Sarcoidosis is characterized by epithelia-cell-rich granulomas, which are non-necrotizing. TB is characterized by caseating granulomas, but the typical manifestations are not always seen in the sample (6), while the sensitivity of the sputum smear and MTB culture is low (7). Many efforts have been made, such as MTB-specific antigens (8) and GeneXpert MTB/RIF (9), to differentiate these two diseases. However, in clinical practice, the differential diagnosis of sarcoidosis and TB is still difficult. Recently, researchers have begun to use omics to identify the similarities and differences between sarcoidosis and TB (10, 11), which could help in the differential diagnosis of the two diseases and in revealing the potential mechanism underlying the diseases.

microRNAs (miRNAs) are a class of endogenous small molecule single-stranded non-coding RNAs that regulate gene expression by translational inhibition and degradation of their target mRNAs at the posttranscriptional level (12), playing an important role in physiological and pathological processes. Aberrant miRNA expression in sarcoidosis (13–15) and TB (16, 17) has been investigated, with most studies exploring the diagnostic performance of miRNA expression in peripheral blood mononuclear cells (13, 14, 17), bronchoalveolar lavage cells (15), urine (16), or serum (18) among individuals with sarcoidosis or TB compared with the healthy controls. In Jie-ru Wang et al.'s study, miR-625-3p in urine showed 98.5% sensitivity and 86.7% specificity for diagnosing pulmonary tuberculosis compared with the healthy control (13). A study conducted by Christian Ascoli et al. demonstrated that a circulating miRNA signature in peripheral blood mononuclear cells, which consist of eight miRNAs (miR-128-3p, miR-22-5p, miR-30e-3p, miR-4306, miR-92a-1-5p, miR-150-3p, miR-6729-5p, and miR-342-5p), showed 68.18% sensitivity and 71.43% specificity compared with a control group excluding patients with active tuberculosis (16). However, the diagnostic performance of miRNA expression in these specimens was not satisfactory, and no study has focused on the differential diagnosis of sarcoidosis and TB using miRNA expression from lymph node. The typical pathological feature of sarcoidosis and TB is the formation of granuloma. Identifying the miRNA profiles of the lymph nodes in individuals with sarcoidosis and in those with TB will help to reveal the differences in the mechanisms between the two diseases.

Therefore, we conducted a prospective study to perform genome-wide miRNA profiling in the lymph nodes of patients with sarcoidosis and TB lymphadenitis (TBLN) as well as in the lymph nodes of controls to identify the differentially expressed miRNAs. The differentially expressed miRNAs were further validated by quantitative real-time PCR to explore the diagnostic value of the miRNAs and the ability of the miRNAs to be used for the differential diagnosis of sarcoidosis and TBLN. Additionally, the correlations between miRNAs and the clinical manifestation of sarcoidosis were examined.

## Materials and Methods

### Subjects

The study was approved by the First Hospital of China Medical University Ethics Board (number: 2019-291-2), and the need for individual patient consent was waived. Thirty-four patients with sarcoidosis were recruited from the First Hospital of China Medical University and were diagnosed according to the international criteria (19). All patients with sarcoidosis were diagnosed due to histological evidence of non-caseating granulomas with typical clinicoradiological images and were followed up for 6 months to exclude other granulomatous diseases. Thirty-three patients with TBLN were recruited from the Chest Hospital of Shenyang; the diagnosis of these patients was based on MTB culture with the biopsy samples or typical histological evidence of caseating necrotizing granuloma. Thirty-three patients with benign thyroid nodules (simple goiter), who were identified by pathological examinations after surgery and were excluded from autoimmune thyroid diseases by serologic examination and thyroid function test, were recruited from the Shengjing Hospital of China Medical University, and these patients acted as the controls. The lymph node tissue samples of the controls (*n* = 3), patients with TBLN (*n* = 3) and patients with sarcoidosis (*n* = 3) were age- and sex-matched (Supplementary Table 1) in the development group and used to perform the miRNA array analyses to determine the significantly differential miRNAs. And the diagnostic performance of the selected miRNAs was verified in the validation group of the controls (*n* = 30), patients with TBLN (*n* = 30) and patients with sarcoidosis (*n* = 31). All study subjects were members of the Chinese Han nationality in northern China. The characteristics of the patients (validation groups) are listed in Table 1.

**Table 1:** The characteristics of the patients in the validation group (mean ± SD).

	**Development group**			**Validation group**		
	**Sarcoidosis (*n* = 3)**	**Tuberculous lymphadenitis (*n* = 3)**	**Control (*n* = 3)**	**Sarcoidosis (*n* = 31)**	**Tuberculous lymphadenitis (*n* = 30)**	**Control (*n* = 30)**
Age (years)	51.00 ± 4.58	36.33 ± 9.29	56.00 ± 9.54	51.94 ± 8.17	38.90 ± 18.80	52.00 ± 13.56
Sex (male: female)	1: 2	1:2	1:2	5: 26	11: 19	5: 25
Radiological stage						
Stage I	2	–	–	12	–	–
Stage II	1	–	–	19	–	–
Definite pulmonary involvement (%)	33.33% (1/3)	33.33% (1/3)	–	61.29% (19/31)	33.33% (10/30)	–
Definite extrapulmonary involvement (%)	33.33% (1/3)	100% (3/3)	–	45.16% (14/31)	86.67% (26/30)	–
BALF						
${\text{CD}}_{4}^{+}$/${\text{CD}}_{8}^{+}$	13.68 ± 2.84	–	–	8.16 ± 6.25	–	–
Lymphocyte (%)	20.00 ± 21.66	–	–	21.67 ± 17.26	–	–
Peripheral blood						
Absolute white blood cell count (× 10^9^/L)	6.59 ± 2.45	7.07 ± 2.35	5.98 ± 2.33	5.45 ± 1.61	6.30 ± 1.69	5.99 ± 1.51
Lymphocyte count (× 10^9^/L)	1.66 ± 0.53	1.50 ± 0.44	2.10 ± 0.53	1.33 ± 0.47	1.71 ± 0.63	1.98 ± 0.45
Lymphocyte (%)	26.10 ± 5.13	22.07 ± 5.09	37.30 ± 7.91	25.16 ± 7.92	27.66 ± 9.33	33.72 ± 5.50
Monocyte (× 10^9^/L)	0.60 ± 0.18	0.50 ± 0.26	0.30 ± 0.10	0.47 ± 0.16	0.55 ± 0.23	0.38 ± 0.11
Monocyte (%)	9.43 ± 2.15	7.00 ± 1.44	5.40 ± 1.57	8.95 ± 2.89	8.84 ± 2.51	6.36 ± 1.37

*SAR, sarcoidosis; TBLN, tuberculous lymphadenitis; Con, control; BALF, bronchoalveolar lavage fluid; SD, standard deviation*.

### Collection and Processing of Samples

The lymph node samples from the patients with sarcoidosis were collected from the intrathoracic lymph node by endobronchial ultrasound-guided transbronchial needle aspiration (EBUS-TBNA). The lymph node samples from the patients with TBLN were collected from the intrathoracic lymph node by EBUS-TBNA or from the cervical lymph node by surgery. The lymph node samples from the control patients were collected from the thyroid by surgery.

### miRNA Microarray

Total RNA including miRNA was isolated from lymph node tissue with an miRNeasy mini kit (QIAGEN, Hilden, Germany) according to manufacturer's protocol. The quality and quantity of total RNA was assessed by spectrophotometry using a NanoDrop 1,000 (Bio Tek Instrument Inc., Winooski, United States). A reverse transcription (RT) reaction was performed using an ABI Veriti Thermal cycler (Applied Biosystems, Foster City, CA, United States), followed by a TaqMan MicroRNA Reverse Transcription kit and Megaplex™ RT Primers (Applied Biosystems). For each reaction, 500 ng of total RNA from the lymph nodes was added to the RT reaction mixture to synthesize single-stranded cDNA. With a TaqMan Low-Density Array Human MicroRNA Array card 1.0, the miRNA profiling assays were performed using an Applied Biosystems 7900HT Fast Real-Time PCR System using the cycling conditions recommended by the manufacturer.

### Global Normalization

The raw cycle threshold (Ct) was calculated using SDS 2.3 and RQ manager 1.2 software, using the automatic baseline and threshold settings (Applied Biosystems, Foster City, CA, United States). The cutoff of the Ct values was set at 35. Global normalization was performed to calculate the mean Ct value of all miRNAs in each sample. Then, the Ct value of each miRNA from the same sample was subtracted from the global mean Ct value to obtain ΔCt. Accordingly, –ΔΔCt= [– (ΔCt_sarcoidosis_ or ΔCt_tuberculosis_-ΔCt_control_)] were calculated based on the obtained ΔCt values.

### Real-Time PCR

The quantification of miRNAs was performed by TaqMan Real-Time PCR according to the manufacturer's protocol. The real-time PCR reactions were carried out using the TaqMan MicroRNA Reverse Transcription kit with specific miRNA TaqMan primers (Applied Biosystems). Real-time PCR was performed with TaqMan® Universal PCR Master Mix and specific probes for TaqMan® MicroRNA (Applied Biosystems). Normalization was performed using endogenous U6 RNA. The expression levels of the miRNAs were calculated using the –ΔCt [–(Ct_miRNA_-Ct_U6_)] method.

### Exploratory Bioinformatics Analysis

To explore the potential role of altered miRNA levels in the pathogenesis of sarcoidosis and TB, the potential target genes of aberrantly expressed miRNAs were predicted by using TargetScan and miRDB on miRWalk3.0. To limit the number of target genes and increase the confidence of the results, the intersection of their predicted target genes was experimentally validated. Kyoto Encyclopedia of Gene and Genomes (KEGG) analysis of predicted target genes was performed to predict the possible pathway involvement and disease-specific biological functions of dysregulated miRNAs among the groups using the DAVID 6.8 website.

### Statistical Analysis

Statistical analysis was performed using SPSS 21 (SPSS Inc., Chicago, IL, United States) and GraphPad Prism 7.00 (GraphPad, La Jolla, CA, United States). A heat map analysis was used to visualize the expression levels of the miRNAs. Student's *t*-test was used to compare differences among the groups. The relationship between the relative expression levels of the miRNAs and clinical parameters were assessed by Spearman correlation coefficients. Receiver operating characteristic (ROC) curves and areas under the ROC curve (AUCs) were performed to assess the diagnostic ability of the miRNAs, and *P* < 0.05 was considered significant.

## Results

### miRNA Microarray Analysis

**Figure 1:**
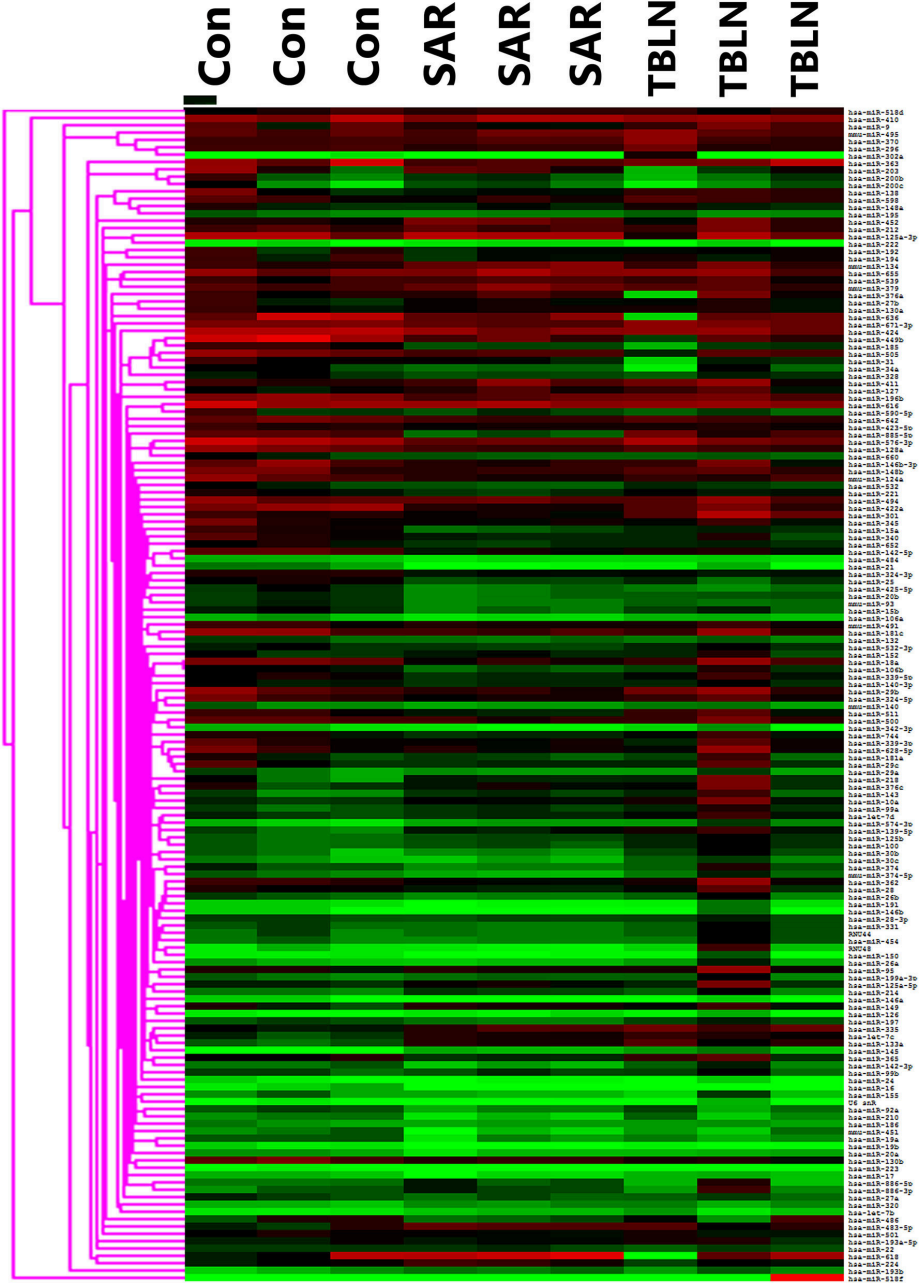
Heat map showing the miRNA profiles of the lymph node tissues in development groups of the control (*n* = 3), sarcoidosis (*n* = 3), and tuberculous lymphadenitis (*n* = 3). SAR, sarcoidosis; TBLN, tuberculous lymphadenitis; Con, control.

**Figure 2:**
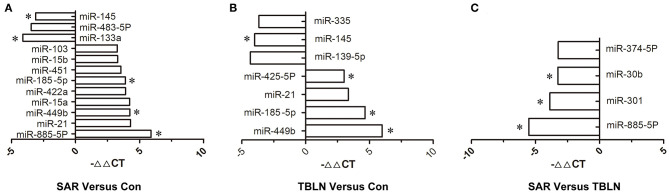
Differentially expressed miRNAs in the lymph nodes of patients between groups in the development group [(|ΔΔCt|≥2) and *P* < 0.05]. The miRNAs to be validated were selected based on the fold change between the groups (|ΔΔCt|≥2 and *P* < 0.05), the *P*-value and the consistency of the miRNA expression in the development group. The -ΔΔCt of the miRNA between groups was shown: **(A)** Between the sarcoidosis group and control group, the -ΔΔCt of miR-885-5P, miR-21, miR-449b, miR-15a, miR-422a, miR-185-5p, miR-451, miR-15b, miR-103, miR-133a, miR-483-5p, and miR-145 was 5.893, 4.291, 4.232, 4.215, 3.902, 3.893, 3.547, 3.284, 3.262, −4.111, −3.453, and −3.117, respectively. **(B)** Between the tuberculous lymphadenitis group and control group, the –ΔΔCt of miR-449b, miR-185-5p, miR-21, miR-425-5P, miR-139-5P, miR-145, and miR-335 was 5.995, 4.661, 3.348, 3.018, −4.330, −3.994, and −3.664, respectively. **(C)** Between the sarcoidosis group and tuberculous lymphadenitis group, the –ΔΔCt of miR-885-5P, miR-301, miR-30b, and miR-374-5P was −5.524, −3.880, −3.256, and −3.229, respectively. For the figure, the *P*-value of all miRNA in the figure were <0.05. *miRNAs selected for validation. SAR, sarcoidosis; TBLN, tuberculous lymphadenitis; Con, control.

The results showed that 197 miRNAs were detected in the controls, 174 miRNAs were detected in the patients with TB and 237 miRNAs were detected in the patients with sarcoidosis (Figure 1). The raw data of miRNA microarray was shown in Supplementary Material. Seventy-five miRNAs were significantly differentially expressed between the patients with sarcoidosis and the controls (*P* < 0.05), twenty-three miRNAs were differentially expressed between the patients with tuberculous lymphadenitis and controls (*P* < 0.05), nineteen miRNAs were significantly differentially expressed between the patients with tuberculous lymphadenitis and the patients with sarcoidosis (*P* < 0.05) and twenty-two miRNAs showed consistent differential expression in patients with either disease compared to the expression levels in the controls (*P* < 0.05) (Supplementary Table 2). Finally, the miRNAs to be validated were selected based on the fold change between the groups [(|ΔΔCt|≥2) and *P* < 0.05], the *P*-value and the consistency of the miRNA expression in the development group (Figure 2).

### Expression of miRNAs in Patients With Sarcoidosis

**Figure 3:**
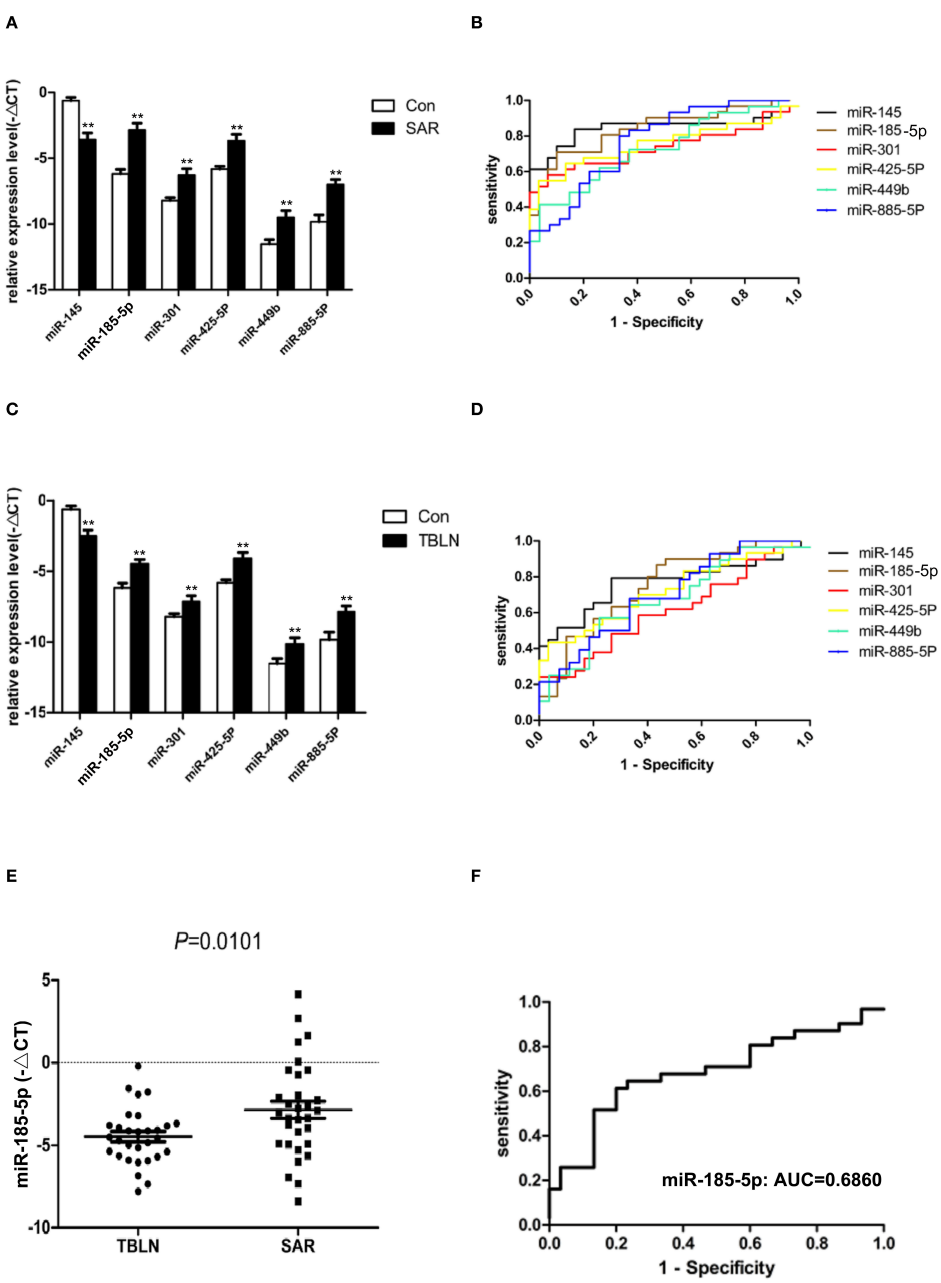
Differentially expressed miRNAs confirmed by quantitative real-time PCR between the groups. Normalization was performed with endogenous U6 RNA. The data are presented as the means ± SEM. Area under the receiver operating characteristic curve (AUC) analyses were performed. **(A)** miR-145, miR-185-5p, miR-301, miR-425-5P, miR-449b, and miR-885-5P were differentially expressed between sarcoidosis and control individuals (*P* < 0.0001, *P* < 0.0001, *P* = 0.0008, *P* = 0.0002, *P* = 0.0018, and *P* < 0.0001, respectively). **(B)** The AUCs of miR-145, miR-185-5p, miR-301, miR-425-5P, miR-449b, and miR-885-5P were 0.8462, 0.8398, 0.7366, 0.7559, 0.7280, and 0.7716, respectively. **(C)** miR-145, miR-185-5p, miR-301, miR-425-5P, miR-449b, and miR-885-5P were differentially expressed between tuberculous lymphadenitis and control individuals (*P* = 0.0002, *P* = 0.0004, *P* = 0.0238, *P* = 0.0006, *P* = 0.0149, and *P* = 0.0045, respectively). **(D)** The AUCs of miR-145, miR-185-5p, miR-301, miR-425-5P, miR-449b, and miR-885-5P were 0.7690, 0.7522, 0.6161, 0.7244, 0.6772, and 0.7050, respectively. **(E)** miR-185-5p in lymph node tissues was differentially expressed between sarcoidosis and tuberculous lymphadenitis (*P* = 0.0101). **(F)** The AUC value of miR-185-5p for the differential diagnosis of sarcoidosis from tuberculous lymphadenitis was 0.6860. **The *P*-value was <0.01 compared to the control group.

Eight miRNAs (miR-30b, miR-133a, miR-145, miR-185-5p, miR-301, miR-425-5P, miR-449b, and miR-885-5P) were subjected to real-time PCR in the validation group of control group (*n* = 30), the tuberculous lymphadenitis group (*n* = 30) and the sarcoidosis group (*n* = 31). The results showed that miR-145 was significantly downregulated in patients with sarcoidosis compared to its expression in the controls (*P* < 0.0001), and miR-185-5p, miR-301, miR-425-5P, miR-449b, and miR-885-5P were significantly upregulated in patients with sarcoidosis compared to their expression in the controls (*P* < 0.0001, *P* = 0.0008, *P* = 0.0002, *P* = 0.0018 and *P* < 0.0001, respectively) (Figure 3A). The ROC curves were analyzed to evaluate the diagnostic value of these miRNAs (Figure 3B). The diagnostic yields were 83.90%, 88%, 90%, 94.40%, 92.30% and 72.70% for miR-145, miR-185-5p, miR-301, miR-425-5P, miR-449b and miR-885-5P, respectively.

### Expression of miRNAs in Patients With TBLN

Interestingly, miR-145 was found to be significantly downregulated in patients with tuberculous lymphadenitis compared to its expression in the controls (*P* = 0.0002). miR-185-5p, miR-301, miR-425-5P, miR-449b, and miR-885-5P were significantly upregulated in patients with tuberculous lymphadenitis compared to their expression in the controls (*P* = 0.0004, *P* = 0.0238, *P* = 0.0006, *P* = 0.0149 and *P* = 0.0045, respectively) (Figure 3C). The ROC curves were also analyzed (Figure 3D). The miRNAs panel showed certain diagnostic power to distinguish sarcoidosis from controls.

### Differential miRNA Expression Between Patients With Sarcoidosis and Patients With Tuberculous Lymphadenitis

Only miR-185-5p was significantly upregulated in patients with sarcoidosis compared to its expression in patients with tuberculous lymphadenitis (*P* < 0.05) (Figure 3E). The AUC of miRNA-185-5p was 0.6860 (Figure 3F), and the sensitivity and specificity values of miR-185-5p were 61 and 80%, respectively, in the differentiation of sarcoidosis from tuberculous lymphadenitis.

### Correlation Between Differentially Expressed miRNAs and the Clinical Parameters of Sarcoidosis

**Figure 4:**
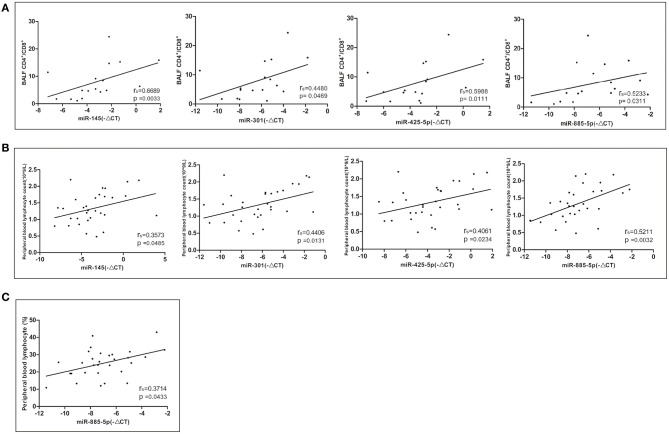
Correlations between the relative expression level of the miRNAs (–ΔCT) and CD4^+^/CD8^+^ lymphocytes in bronchoalveolar lavage fluid (BALF) and peripheral blood. Spearman correlation coefficients were used to evaluate the strength of the relationships (*P* < 0.05 was considered significant). **(A)** Correlation between the relative expression of miR-145, miR-301, miR-425-5P, and miR-885-5P and CD4^+^/CD8+ lymphocytes in BALF. **(B)** Correlation between the relative expression of miR-145, miR-301, miR-425-5P, and miR-885-5P and the peripheral blood lymphocyte count. **(C)** Correlation between the relative expression of miR-885-5P and peripheral blood lymphocytes (%).

The relative expression levels of miR-145, miR-301, miR-425-5P, and miR-885-5P were positively correlated with the ratio of CD4+/CD8+ lymphocytes in bronchoalveolar lavage fluid (BALF) (Figure 4A). The relative expression levels of miR-145, miR-301, miR-425-5P, and miR-885-5P were significantly correlated with the peripheral blood lymphocyte count (Figure 4B). There was a significant positive correlation between the relative expression of miR-885-5P and peripheral blood lymphocyte percentage (Figure 4C). There was no significant correlation between the expression levels of differentially expressed miRNAs and the stage, sex or age of the patients with sarcoidosis (data not shown).

### Exploratory Bioinformatics Analysis

**Figure 5:**
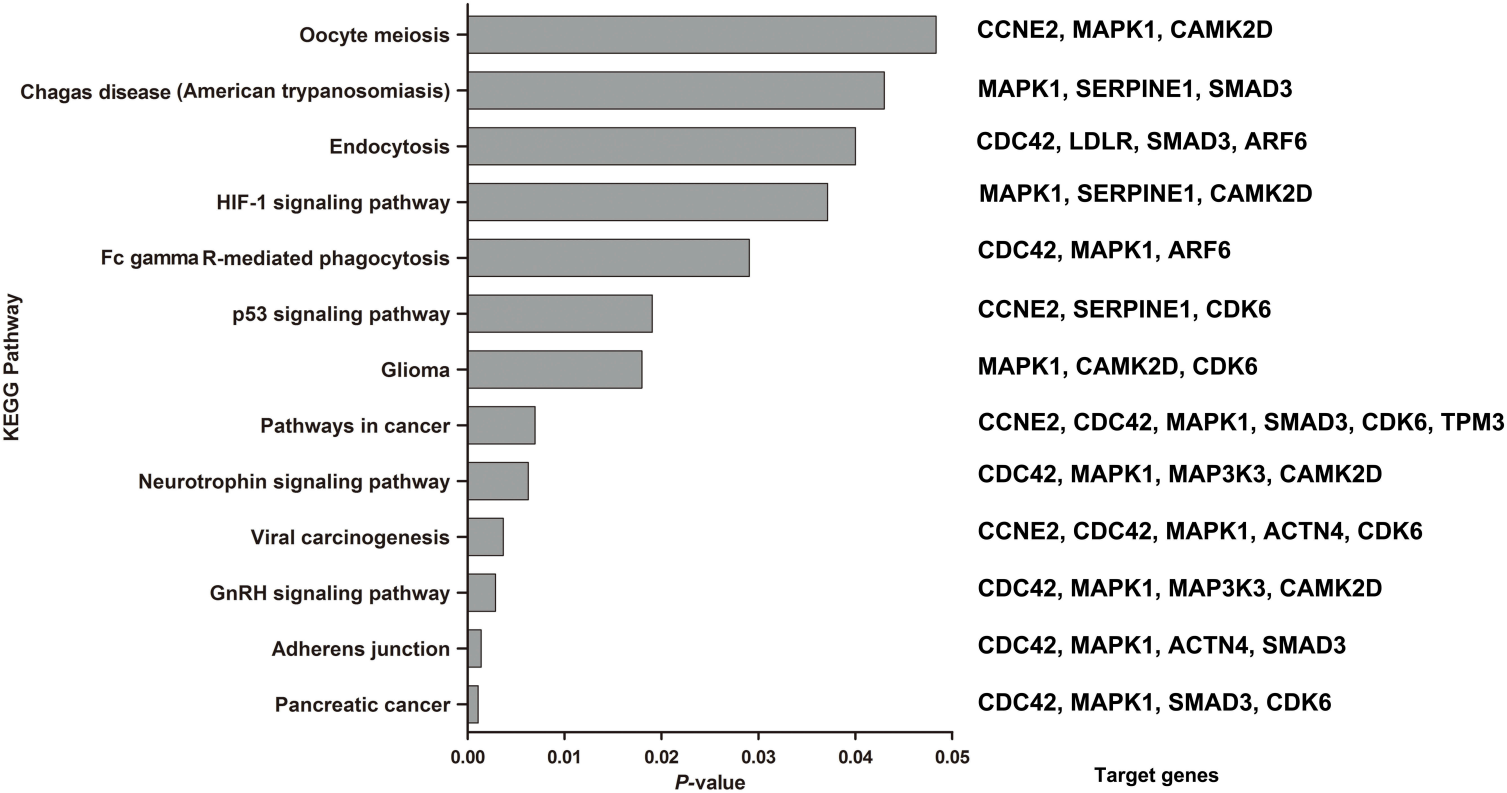
KEGG pathways enriched by the 6 miRNAs by the miRNA-target algorithm. Significance was established with a *P*-value threshold of <0.05. On the right were the target genes involved in the corresponding KEGG pathway in our analysis.

The combination of these 6 miRNAs (miR-145, miR-185-5p, miR-301, miR-425-5P, miR-449b, and miR-885-5P) resulted in forty-nine targeted genes based on functional enrichment analysis of miRNA–gene interactions obtained from the KEGG (Supplementary Table 3) Seventeen enriched pathways containing twenty-three targeted genes were identified by KEGG analysis, in which a total of 13 enriched pathways met the redetermined level of significance (*P* < 0.05) (Figure 5). The corresponding targeted gene were shown in the Figure 5 and Supplementary Table 4.

Among the significantly enriched pathways, the majority of pathways were involved in molecular interaction and reaction networks pertaining to human diseases (38.46%, mainly cancers), environmental information processing (signal transduction, 30.77%), and cellular processes (30.77%). The most significantly identified enriched pathway were about the pancreatic cancer (*P* = 0.0011), which four target genes (CDC42, MAPK1, SMAD3, and CDK6) were predicted to be involved in. And the secondary was about the adherens junction (*P* = 0.0014), which four target genes (CDC42, MAPK1, ACTN4, and SMAD3) were predicted to be involved in. In addition, the HIF-1 (*P* = 0.0371), neurotrophin (*P* = 0.0063) and p53 signaling pathways (*P* = 0.019) were also predicted to be significantly enriched pathways.

## Discussion

There is a common view that the exclusion of alternative diseases can be used for the differential diagnosis of sarcoidosis (20). However, the differential diagnosis of sarcoidosis and TB is challenging considering the profound similarities in clinical symptoms and histopathological characteristics. Hence, this study investigated the value of miRNAs in the differential diagnosis of sarcoidosis and TBLN. In this study, six miRNAs were differentially expressed between patients with sarcoidosis and controls, demonstrating a high diagnostic value for sarcoidosis. The AUC of miR-145 in sarcoidosis was 0.8462, and the sensitivity and specificity were 84 and 83%, respectively.

Many studies have been conducted to discover a biomarker for sarcoidosis. Angiotensin-converting enzyme was once highly promising as a circulatory biomarker, but its poor sensitivity (41.1%) and unsatisfied specificity (89.9%) were demonstrated in a recent study (21). As key regulators of gene expression by silencing or destabilizing their target RNAs, miRNAs have attracted increasing attention for the diagnosis of sarcoidosis. Consistent with our study, previous studies demonstrated significant differences in miRNA expression between sarcoidosis patients and healthy individuals (10, 13–15, 22–24). Ascoli et al. (13) reported a diagnostic accuracy of 74.8%, a sensitivity of 68.18% and a specificity of 71.43% for the combination of eight differentially expressed miRNAs in peripheral blood mononuclear cells. Nevertheless, the diagnostic performance of circulatory miRNAs is not sufficient for either the differential diagnosis of sarcoidosis from other granulomatous diseases such as TBLN in the real world or to prevent the need for invasive procedures for histopathological examination.

A previous study conducted by Jeroen Maertzdorf and colleagues suggested that four miRNAs (has-miRNA-182, miR-355, miR-15b, miR-340) in blood were significantly different between subjects with sarcoidosis and those with TB using microarray (10). Similar to the results of our study, the authors concluded that there were remarkable differences in miRNA levels between diseased individuals (sarcoidosis or TB) and healthy controls, and there was high similarity in miRNA profiles between patients with sarcoidosis and those with TB. It is worth noting that the four differentially expressed miRNAs in the peripheral blood did not overlap with the six differentially expressed miRNAs in the lymph nodes found in our study. This difference may be due to the tissue or cycle specificity of the miRNAs, representing the distinct characteristic of disease manifestation between the local tissues and overall circulation, which has related biological significance (24).

miRNAs in the lymph nodes display similar expression patterns between sarcoidosis and TB, and the same six miRNAs were differentially expressed in patients with TB compared to their expression levels in the controls. Studies have shown that sarcoidosis and TB show similar expression in both genomics and transcriptomics (10, 25), which can be explained by the similarity in the pathology and pathogenesis between sarcoidosis and TB. Therefore, the efficacy of miRNAs for the diagnosis of only sarcoidosis or TB requires validation between sarcoidosis and TB, especially in countries with high morbidity of TB.

miR-185-5p is differentially expressed between individuals with sarcoidosis and those with TB. The sensitivity and specificity for the diagnosis of sarcoidosis were 61 and 80%, respectively, indicating that miR-185-5p can be used as an auxiliary biomarker for the differential diagnosis of the two diseases. Compared with the diagnostic value of GeneXpert MTB/RIF in the lymph node tissue for intrathoracic sarcoidosis and intrathoracic lymph node TB (sensitivity of 49.1% and specificity of 97.9%) (9), the specificity is higher, and the combination of the two methods may help to distinguish sarcoidosis from TB. In our study, the six differentially expressed miRNAs (miR-145, miR-185-5p, miR-301, miR-425-5P, miR-449b, and miR-885-5P) found in our studies showed good diagnostic yield (yields were 83.90%, 88%, 90%, 94.40%, 92.30%, and 72.70% for miR-145, miR-185-5p, miR-301, miR-425-5P, miR-449b, and miR-885-5P, respectively) for sarcoidosis when the enrolled sarcoidosis cases were either stage I or stage II. Considering the fact that the enlargement of hilar lymph nodes occurs in the stage I or stage II of Sarcoidosis and that the EBUS-TBNA was usually used to obtain biopsies in patients with undiagnosed enlarged mediastinal lymph node, the mentioned six miRNAs may be useful in the early stage diagnosis.

The ratio of CD4+/CD8+ lymphocytes in BALF is often elevated in patients with sarcoidosis. As markers for the diagnosis of sarcoidosis, miR-145, miR-301, miR-425-5P, and miR-885-5P were significantly positively correlated with the ratio of CD4+/CD8+ lymphocytes in the BALF of patients with sarcoidosis, suggesting a potential link between the expression of these miRNAs and CD4+ lymphocyte activation and aggregation in sarcoidosis. The relative expression levels of miR-885 exhibited a positive correlation with the proportion of peripheral blood lymphocytes in patients with sarcoidosis; therefore, miR-885 may participate in the regulation of peripheral blood lymphocytes. However, the specific mechanism needs to be explored in further research.

To understand the biological functions of six miRNAs that are differentially expressed in sarcoidosis patients, targeted gene prediction and KEGG pathway analysis were performed. Sarcoidosis is characterized by extensive local inflammation and granuloma formation in different organs, especially in lung. Among the identified pathways, the HIF-1 (26, 27) and neurotrophin (28) signaling pathways, whose corresponding target genes were MAPK1, SERPINE1 and CAMK2D for HIF-1 signaling pathways and CDC42, MAPK1, MAP3K3, and CAMK2D for neurotrophin signaling pathways in our analysis, have been implicated in the inflammation pathogenesis of sarcoidosis according to previous studies. The HIF-1 signaling pathway was proven to regulate Th1/Th17 mediated inflammation in sarcoidosis (27) and be linked to imbalance of ${\text{CD}}_{4}^{+}$/${\text{CD}}_{8}^{+}$ in BALF and acknowledged negative prognostics (26), consistence with the result that the expression of four miRNA had moderate correlation with the ratio of ${\text{CD}}_{4}^{+}$/${\text{CD}}_{8}^{+}$. Our result provided supplementary evidence about how miRNAs participated in the regulate the pathogenesis of sarcoidosis. P53, HIF-1, and Fc-gamma-receptor-mediated phagocytic and endocytic signaling processes have been confirmed in patients with sarcoidosis in previous studies (26, 29–32). Similarly, these pathways have also been conformed in patients with TB (33–36), which further illustrates the similarity in the pathogenesis of sarcoidosis and TB. In addition, the KEGG analysis revealed a variety of tumor-associated signaling pathways involving the miRNA target genes, which may be related to the cell proliferation of sarcoidosis and tumors.

In this study, the spectrum of miRNA expression in lymph node lesions of patients with sarcoidosis and patients with TB was first analyzed, which helped to better understand these diseases. However, this was a single-center study based on a small sample size, and we validated only a portion of the differentially expressed miRNAs. For sarcoidosis patients, only Phase I-II patients were enrolled; therefore, the results cannot reflect the entire process of sarcoidosis. Further investigations of the miRNA expression of patients with sarcoidosis and patients with TB are warranted. Although the potential functions of miRNAs in sarcoidosis and TB were explored through bioinformatics, the specific mechanisms need to be explored in future research. In addition, as the result of our study and the previous study (37), both tuberculosis and sarcoidosis demonstrated highly similar level of miRNA expression. This finding is consistent with the view that miRNAs are primarily responsible for fine tuning of responses rather than on/off switch signals. This could be the reason of limited different diagnosed performance of miRNAs as independent biomarker. But miR-185-5p still works for helping differential diagnose between TBLN and sarcoidosis. A further study should be conducted to get a new way with higher sensitivity and specificity by combining miR-185-5p in the lymph nodes and other parameters.

In summary, miRNAs in lymph nodes display similar expression patterns between patients with sarcoidosis and patients with TBLN, which were experimentally selected. miR-185-5p in the lymph nodes can be used as a marker for the differential diagnosis of sarcoidosis and TBLN.

## Data Availability Statement

The data has been uploaded to ArrayExpress–E-MTAB-9518.

## Ethics Statement

The studies involving human participants were reviewed and approved by the First Hospital of China Medical University Ethics Board. Written informed consent for participation was not required for this study in accordance with the national legislation and the institutional requirements.

## Author Contributions

GH and R-qQ: conception and design. GH and JK: administrative support. C-jY and W-xX: provision of study materials or patients. GH, R-qQ, YY, WL, QZ, C-jY, and W-xX: collection and assembly of data. WL, YY, R-qQ, QZ, C-jY, and W-xX: data analysis and interpretation. All authors: manuscript writing and final approval of manuscript.

## Conflict of Interest

The authors declare that the research was conducted in the absence of any commercial or financial relationships that could be construed as a potential conflict of interest.
