# Supplementary material for: Distinct miRNA Gene Expression Profiles Among the Nodule Tissues of Lung Sarcoidosis, Tuberculous Lymphadenitis and Normal Healthy Control Individuals
Source: Front Med (Lausanne). 2020 Oct 16;7:527433. doi: 10.3389/fmed.2020.527433 (PMC7596360; doi:10.3389/fmed.2020.527433)
Supplement: Supplementary file 1 [file Data_Sheet_1.docx]

Supplementary Material

# Supplementary Tables

**Table S1.** Demographic characteristics of the patients (Screening groups)

|  | SAR(n=3) | | | TBLN(n=3) | | | Con(n=3) | | |
| --- | --- | --- | --- | --- | --- | --- | --- | --- | --- |
| Number | SAR1 | SAR2 | SAR3 | TBLN1 | TBLN2 | TBLN3 | Con1 | Con2 | Con3 |
| Sex(F/W) | W | W | F | W | W | F | W | W | F |
| Age(years) | 32 | 34 | 40 | 32 | 34 | 40 | 32 | 34 | 40 |

Abbreviations: SAR, sarcoidosis; TBLN, tuberculous lymphadenitis; Con, control.

**Table S2.** Differentially expressed miRNAs in the lymph nodes of patients with SAR, TBLN and Con(*P*<0.05)

| **SAR Versus Con** | | | | **TBLN Versus Con** | | | | **SAR Versus TBLN** | | |
| --- | --- | --- | --- | --- | --- | --- | --- | --- | --- | --- |
| **Has-miRNA** | **-△△CT** | ***P* Value** | **Has-miRNA** | | **-△△CT** | ***P* Value** | **Has-miRNA** | | **-△△CT** | ***P* Value** |
| \| hsa-let-7b-002619 \| \| --- \| \| hsa-let-7c-000379 \| \| hsa-miR-103-000439 \| \| hsa-miR-106a-  002169 \| \| hsa-miR-106b-  000442 \| \| hsa-miR-10a-000387 \| \| hsa-miR-125a-5p-002198 \| \| hsa-miR-125b-000449 \| \| hsa-miR-128a-002216 \| \| hsa-miR-130b-000456 \| \| hsa-miR-133a-002246 \| \| hsa-miR-139-5p-002289 \| \| hsa-miR-142-3p-000464 \| \| hsa-miR-142-5p-002248 \| \| hsa-miR-145-002278 \| \| hsa-miR-146a-000468 \| \| hsa-miR-150-000473 \| \| hsa-miR-15a-000389 \| \| hsa-miR-15b-000390 \| \| hsa-miR-16-000391 \| \| hsa-miR-185-002271 \| \| hsa-miR-18a-002422 \| \| hsa-miR-190-000489 \| \| hsa-miR-191-002299 \| \| hsa-miR-193b-002367 \| \| hsa-miR-196b-002215 \| \| hsa-miR-197-000497 \| \| hsa-miR-19a-000395 \| \| hsa-miR-19b-000396 \| \| hsa-miR-20b-001014 \| \| hsa-miR-210-000512 \| \| hsa-miR-21-000397 \| \| hsa-miR-214-002306 \| \| hsa-miR-221-000524 \| \| hsa-miR-224-002099 \| \| hsa-miR-25-000403 \| \| hsa-miR-26b-000407 \| \| hsa-miR-28-000411 \| \| hsa-miR-320-002277 \| \| hsa-miR-324-3p-002161 \| \| hsa-miR-328-000543 \| \| hsa-miR-335-000546 \| \| hsa-miR-339-5p-002257 \| \| hsa-miR-340-002258 \| \| hsa-miR-362-001273 \| \| hsa-miR-365-001020 \| \| hsa-miR-374-000563 \| \| hsa-miR-384-000574 \| \| hsa-miR-422a-  002297 \| \| hsa-miR-424-000604 \| \| hsa-miR-425-5p-001516 \| \| hsa-miR-449b-001608 \| \| hsa-miR-450b-3p-002208 \| \| hsa-miR-452-002329 \| \| hsa-miR-483-5p-002338 \| \| hsa-miR-484-001821 \| \| hsa-miR-502-001109 \| \| hsa-miR-502-3p-002083 \| \| hsa-miR-505-002089 \| \| hsa-miR-511-001111 \| \| hsa-miR-518e-002395 \| \| hsa-miR-576-3p-002351 \| \| hsa-miR-625-002431 \| \| hsa-miR-642-001592 \| \| hsa-miR-652-002352 \| \| hsa-miR-671-3p-002322 \| \| hsa-miR-708-002341 \| \| hsa-miR-744-002324 \| \| hsa-miR-885-5p-002296 \| \| hsa-miR-92a-000431 \| \| mmu-miR-124a-001182 \| \| mmu-miR-134-001186 \| \| mmu-miR-374-5p-001319 \| \| mmu-miR-451-001141 \| \| mmu-miR-93-001090 \| | \| -1.43 \| \| --- \| \| -2.78 \| \| 3.26 \| \| 1.55 \| \| 2.89 \| \| -1.42 \| \| -1.45 \| \| -1.15 \| \| 2.23 \| \| 1.64 \| \| -4.11 \| \| -2.79 \| \|  \| \| 2.55 \| \| 2.88 \| \| -3.12 \| \| 2.87 \| \| 0.88 \| \| 4.22 \| \| 3.28 \| \| 2.84 \| \| 3.89 \| \| 2.9 \| \| 2.57 \| \| 1.22 \| \| -2.11 \| \| 1.56 \| \| 1.54 \| \| 2.91 \| \| 2.21 \| \| 2.55 \| \| 2.88 \| \| 4.29 \| \| -2.11 \| \| 1.91 \| \| -1.77 \| \| 2.9 \| \| 1.56 \| \| 1.57 \| \| -1.45 \| \| 1.55 \| \| 1.53 \| \| -2.77 \| \| 1.91 \| \| 2.55 \| \| 1.9 \| \| 1.88 \| \| 2.23 \| \| 2.68 \| \| 3.9 \| \| 1.55 \| \| 2.91 \| \| 4.23 \| \| 1.57 \| \| -2.79 \| \| -3.45 \| \| 1.23 \| \| 1.54 \| \| 1.88 \| \| 1.55 \| \| 2.56 \| \| 1.55 \| \| 2.56 \| \| 2.57 \| \| 1.55 \| \| 1.9 \| \| 1.22 \| \| 2.23 \| \| 1.88 \| \| 5.89 \| \| 2.23 \| \| 2.58 \| \| -2.44 \| \| 2.23 \| \| 3.55 \| \| 2.91 \| | \| 0.002 \| \| --- \| \| 0.011 \| \| 0.022 \| \| 0.045 \| \| 0.005 \| \| 0.013 \| \| 0.023 \| \| 0.026 \| \| 0.013 \| \| 0.041 \| \| 0.001 \| \| 0.031 \| \| 0.015 \| \| 0.005 \| \| 0.002 \| \| 0.017 \| \| 0.036 \| \| 0.003 \| \| 0.007 \| \| 0.024 \| \| 0.003 \| \| 0.005 \| \| 0.009 \| \| 0.014 \| \| 0.040 \| \| 0.037 \| \| 0.011 \| \| 0.050 \| \| 0.020 \| \| 0.008 \| \| 0.009 \| \| 0.003 \| \| 0.002 \| \| 0.029 \| \| 0.043 \| \| 0.001 \| \| 0.041 \| \| 0.011 \| \| 0.016 \| \| 0.001 \| \| 0.042 \| \| 0.009 \| \| 0.005 \| \| 0.038 \| \| 0.003 \| \| 0.016 \| \| 0.027 \| \| 0.008 \| \| 0.001 \| \| 0.023 \| \| 0.019 \| \| 0.014 \| \| 0.046 \| \| 0.006 \| \| 0.024 \| \| 0.015 \| \| 0.042 \| \| 0.003 \| \| 0.044 \| \| 0.013 \| \| 0.040 \| \| 0.009 \| \| 0.024 \| \| 0.010 \| \| 0.028 \| \| 0.003 \| \| 0.03 \| \| 0.035 \| \| <0.001 \| \| 0.019 \| \| 0.035 \| \| 0.009 \| \| 0.014 \| \| 0.023 \| \| 0.001 \| | \| hsa-let-7c-000379 \| \| --- \| \| hsa-miR-100-000437 \| \| hsa-miR-125b-000449 \| \| hsa-miR-128a-002216 \| \| hsa-miR-130b-000456 \| \| hsa-miR-133a-002246 \| \| hsa-miR-139-5p-002289 \| \| hsa-miR-142-5p-002248 \| \| hsa-miR-145-002278 \| \| hsa-miR-15a-000389 \| \| hsa-miR-16-000391 \| \| hsa-miR-185-002271 \| \| hsa-miR-19a-000395 \| \| hsa-miR-20b-001014 \| \| hsa-miR-21-000397 \| \| hsa-miR-223-002295 \| \| hsa-miR-25-000403 \| \| hsa-miR-324-3p-002161 \| \| hsa-miR-335-000546 \| \| hsa-miR-424-000604 \| \| hsa-miR-425-5p-001516 \| \| hsa-miR-449b-001608 \| \| hsa-miR-484-001821 \| \| mmu-miR-93-001090 \| | | \| -2.98 \| \| --- \| \| -2.65 \| \| -2.67 \| \| 2 \| \| 2.7 \| \| -4.66 \| \| -4.33 \| \| 2.33 \| \| -3.99 \| \| 2.35 \| \| 2.31 \| \| 4.66 \| \| 2.02 \| \| 1.34 \| \| 3.35 \| \| 2.67 \| \| 2.7 \| \| 1.68 \| \| -3.66 \| \| 1.67 \| \| 3.02 \| \| 5.99 \| \| 1.01 \| \| 2.03 \| | \| 0.014 \| \| --- \| \| 0.009 \| \| 0.010 \| \| 0.034 \| \| 0.020 \| \| 0.006 \| \| 0.018 \| \| 0.014 \| \| 0.009 \| \| 0.011 \| \| 0.035 \| \| 0.042 \| \| 0.019 \| \| 0.016 \| \| 0.049 \| \| 0.001 \| \| 0.040 \| \| 0.002 \| \| 0.004 \| \| 0.033 \| \| 0.015 \| \| 0.026 \| \| 0.030  0.005 \| | \| hsa-miR-138-002284 \| \| --- \| \| hsa-miR-885-5p-002296 \| \| hsa-miR-106a-002169 \| \| hsa-miR-101-002253 \| \| hsa-miR-197-000497 \| \| hsa-miR-15a-000389 \| \| hsa-miR-30b-000602 \| \| hsa-miR-301-000528 \| \| hsa-miR-221-000524 \| \| mmu-miR-93-001090 \| \| hsa-miR-30c-000419 \| \| hsa-miR-100-000437 \| \| mmu-miR-374-5p-001319 \| \| hsa-miR-9-000583 \| \| hsa-miR-125b-000449 \| \| hsa-miR-224-002099 \| \| hsa-miR-532-001518 \| \| hsa-miR-17-002308 \| \| hsa-miR-671-3p-002322 \| | | \| -2.21 \| \| --- \| \| -5.52 \| \| -1.21 \| \| -1.16 \| \| -2.19 \| \| -1.87 \| \| -3.26 \| \| -3.88 \| \| -1.23 \| \| -0.88 \| \| -2.89 \| \| -1.88 \| \| -3.23 \| \| -2.56 \| \| -1.52 \| \| 2.46 \| \| -1.23 \| \| -1.56 \| \| -1.21 \| | \| 0.001 \| \| --- \| \| 0.004 \| \| 0.008 \| \| 0.009 \| \| 0.015 \| \| 0.016 \| \| 0.021 \| \| 0.021 \| \| 0.022 \| \| 0.029 \| \| 0.032 \| \| 0.032 \| \| 0.033 \| \| 0.039 \| \| 0.040 \| \| 0.040 \| \| 0.044 \| \| 0.045 \| \| 0.048 \| |

Abbreviations: SAR=sarcoidosis; TBLN=tuberculous lymphadenitis; Con=control.

**Table S3**. Targeted genes of differentially expressed miRNA.

| miRNA | miR-145 | miR-185 | miR-301 | miR-425-5P | miR-449b | miR-885-5P |
| --- | --- | --- | --- | --- | --- | --- |
| genesymbol | ABRACL  ARF6  CBFB  CLINT1  ERG  FSCN1  MAP3K3  MYO6  MYRF  NEDD9  PLAGL2  RREB1  RTKN  SERPINE1  SMAD3  TPM3 | ABCG4  ACTN4  CAMK2D  CDC42  EMC10  LPCAT3  PBX2  SF1  SLC8A1  UHMK1 | PTPRG  NUS1  SALL3  CHIC1  ZNF800  RAB14  MAPK1  NIPA1  LDLR  BTBD3  CDK19  NR3C2  DDX6  FXR1  ARL6IP1 | CREBZF | BCL2L13  CCNE2  CDK6  GMFB  LDHA  PPP1R11  TSN | - |

Note: Predicted targeted genes of differentially expressed miRNA respectively. the intersection of target genes, which were experimentally validated according to previous published studies, predicted by TargetScan and miRDB.

**Table S4** KEGG pathways analyzed by the miRNA-target algorithm and the corresponding Targeted genes.

| Category | Term | P Value | Target gene |
| --- | --- | --- | --- |
| hsa05212 | Pancreatic cancer | 0.0011 | CDC42, MAPK1, SMAD3, CDK6 |
| hsa04520 | Adherens junction | 0.0014 | CDC42, MAPK1, ACTN4, SMAD3 |
| hsa04912 | GnRH signaling pathway | 0.0029 | CDC42, MAPK1, MAP3K3, CAMK2D |
| hsa05203 | Viral carcinogenesis | 0.0037 | CCNE2, CDC42, MAPK1, ACTN4, CDK6 |
| hsa04722 | Neurotrophin signaling pathway | 0.0063 | CDC42, MAPK1, MAP3K3, CAMK2D |
| hsa05200 | Pathways in cancer | 0.007 | CCNE2, CDC42, MAPK1, SMAD3, CDK6, TPM3 |
| hsa05214 | Glioma | 0.018 | MAPK1, CAMK2D, CDK6 |
| hsa04115 | p53 signaling pathway | 0.019 | CCNE2, SERPINE1, CDK6 |
| hsa04666 | Fc gamma R-mediated phagocytosis | 0.0291 | CDC42, MAPK1, ARF6 |
| hsa04066 | HIF-1 signaling pathway | 0.0371 | MAPK1, SERPINE1, CAMK2D |
| hsa04144 | Endocytosis | 0.04 | CDC42, LDLR, SMAD3, ARF6 |
| hsa05142 | Chagas disease (American trypanosomiasis | 0.043 | MAPK1, SERPINE1, SMAD3 |
| hsa04114 | Oocyte meiosis | 0.0483 | CCNE2, MAPK1, CAMK2D |
| hsa04110 | Cell cycle | 0.0589 | CCNE2, SMAD3, CDK6 |
| hsa04261 | Adrenergic signaling in cardiomyocytes | 0.0711 | MAPK1, CAMK2D, TPM3 |
| hsa05161 | Hepatitis B | 0.0775 | CCNE2, MAPK1, CDK6 |
| hsa05216 | Thyroid cancer | 0.0889 | MAPK1, TPM3 |
