# Supplementary material for: Distinct miRNA Gene Expression Profiles Among the Nodule Tissues of Lung Sarcoidosis, Tuberculous Lymphadenitis and Normal Healthy Control Individuals
Source: Front Med (Lausanne). 2020 Oct 16;7:527433. doi: 10.3389/fmed.2020.527433 (PMC7596360; doi:10.3389/fmed.2020.527433)
Supplement: Supplementary file 2 [file Table_5.docx]

| **Assay ID** | **Assay Name** | **miRBase ID** | **miRBase Accession Number** | **miRBase Alias** | **Target Sequence** |
| --- | --- | --- | --- | --- | --- |
| **002271** | **hsa-miR-185** | bta-miR-185::cfa-miR-185::cgr-miR-185-5p::ggo-miR-185::  **hsa-miR-185-5p**  ::mml-miR-185::mmu-miR-185-5p::ppy-miR-185::ptr-miR-185::rno-miR-185-5p::ssc-miR-185 | MIMAT0000214::  MIMAT0000455::  MIMAT0000862::  MIMAT0006219::  MIMAT0006660::  MIMAT0007759::  MIMAT0008053::  MIMAT0009247::  MIMAT0015781::  MIMAT0023811::  MIMAT0024086 | hsa-miR-185(17)  mmu-miR-185(17)::rno-miR-185(18) | UGGAGAGAAAGGCAGUUCCUGA |

**Table S5. The detail information of miRNA-185 in Our miRNA microarray analysis.**
